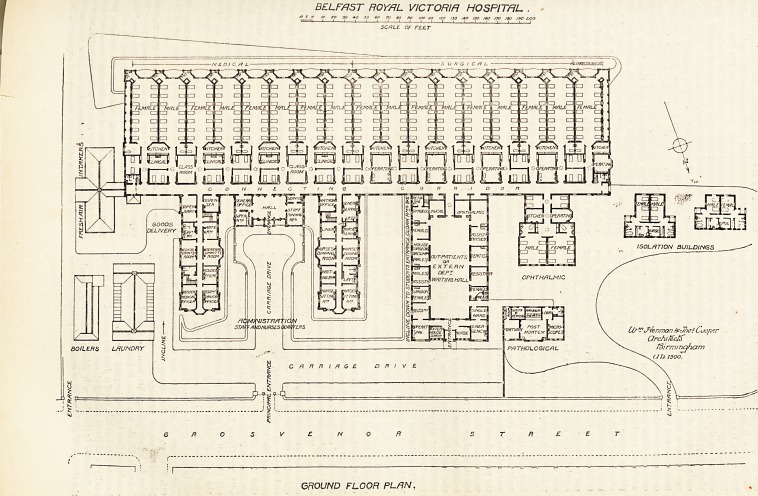# The Royal Victoria Hospital, Belfast

**Published:** 1901-06-22

**Authors:** 


### THE ROYAL VICTORIA HOSPITAL, BELFAST.

The lamented death of Queen Victoria, to whose memory this new hospital at Belfast is dedicated, necessitated the postponement of the ceremony of laying the foundation-stone, which was to have been performed by the Prince and Princess of Wales on April 18, but His Majesty the King has graciously expressed his desire to lay the stone at a later date, so that Belfast will yet be honoured by a Royal visit for this purpose.

There is so much that is novel about this building that the plans which we to-day publish deserve the most careful consideration by hospital architects and hospital managers. They may, in fact, be taken as representing the logically-complete embodiment of the new ideas as to hospital construction which have sprung up since the practicability of mechanical ventilation was demonstrated, and any estimate of their fitness must involve a consideration of the "plenum" system of heating and ventilation, which indeed forms the key to the whole. Some who condemn the system will perhaps condemn, off-hand, the plans as well. That, however, is not the view we take of the position. In considering these plans the question should rather be—how far they utilise the advantages and minimise the evils inherent in the system adopted, and with that view before us, it may be well to say a few words as to some of the difficulties of hospital ventilation.

As everyone knows the forces at work in producing "natural" ventilation are first the wind, which, although very efficient, is an extremely fickle and unreliable one; and secondly, gravitation, the motive power of which is brought into play for ventilating purposes by the difference in the temperature, and therefore in the weight of the air within and outside the building. Except for the influence of the wind, natural ventilation then depends entirely upon differences in temperature, and thus it tends to run to excess in cold weather when they are extreme, and when fires are lighted and chimneys are in full blast, and to stagnation in hot weather when the air within is practically of the same temperature as that outside. Hence the necessity of constant watchfulness and frequent readjustment of the means employed if uniformity of ventilation on the "natural" system is to be obtained. But there are architectural difficulties besides those of mere supervision. When ventilation goes by windows, it is essential that regard should be paid to the condition of the air without, as well as the air within, and such considerable open spaces have to be provided between the pavilion, that a large hospital must either be spread over an inordinately large site, or must be built in many stories, either of which tends to complicate administration. Another trouble in "natural" ventilation has to be mentioned, viz., that of "draughts." Under ordinary circumstances these can generally be avoided by the exercise of care. But in very cold weather it is quite impossible to get the full supply of air (a minimum of 3,000 cubic feet per patient per hour) through the wards without causing discomfort and even danger, unless means be taken to warm the air as it comes in—an affair which involves a somewhat expensive arrangement of hot pipes, the maintenance of which at a proper temperature is not always attained with as great a degree of accuracy as might be wished. To obviate these difficulties various schemes of artificial ventilation have been proposed, among which the plenum system stands pre-eminent from the fact that, as carried out of late years, it concentrates in one complete mechanically-driven installation all the arrangements required for the warming and ventilation of the whole of a building; and it is according to the degree to which the architects of the Belfast Victoria Hospital have succeeded in taking advantage of the benefits offered by this system and avoiding such evils as may be inherent in it, that their plan must be judged.

What, then, are the advantages offered by the system, and what its disadvantages? The advantages claimed by its advocates are that it secures an equable temperature and continual change of air throughout every portion of the building without draughts; that, as there are no fire-places or heating appliances in the wards, noise and dirt caused by making up fires and carrying coal about are entirely avoided; and especially and above all that the amount of air distributed and made to go through the wards is entirely under the control of the engineer, and entirely independent of the state of the weather or the direction of the wind.

On the other hand, the drawbacks are first the question of expense. This we can have no doubt has hitherto been somewhat heavy, and one of the most important questions which it is hoped that the Belfast Hospital will solve is, What is the expense under the most favourable conditions? Then there is the question of the effect upon the health and stamina of those who live under "plenum" conditions. Some of the advocates of mechanical ventilation will probably cry out that we are quite behind the times in suggesting any doubt on the subject. Still, the question has to be faced. It is quite clear that the inhabitants of temperate climes do not naturally live under conditions of equability of temperature in any way approaching those which are provided in a "plenum" hospital, and in view of the results which have of late followed the adoption of the open-air plan of treatment in tuberculosis and other diseases, it is by no means certain that equability of temperature is a thing so greatly to be desired as some have imagined. The proof of the pudding, in this as in so many other cases, lies in the eating, and we have to confess that we do not yet know what is the effect upon the organism of the prolonged abolition of those diurnal changes in man's surroundings which are natural to him. The next point is really a corollary of the last. Whatever the results upon the health may be, no one can doubt that the pleasantest effect in house warming is produced when the house itself—the walls, &c.—are warm and the air circulating therein is "fresh"—that is to say, is rather cool than otherwise. Herein lies the advantage of radiant heat over heated air, of open fires over hot-water pipes. Now with the plenum system in its recent developments all loss of heat through windows, &c., has to be made good by such heat as is brought in by the air of ventilation, and in winter, when this heat loss is considerable, either a very excessive amount of air must be made to pass through the wards or else this air must enter the wards at a a temperature higher than the one fixed as the desirable one. It is, then, important that the plan of the building should be such as to obviate as far as possible the necessity for employing such highly-heated air. Finally there is an objection which is made much of by some, and certainly must be made a good deal of by those who have experienced the vagaries of certain electric lighting companies, and that is the ever present possibility of the machinery coming to a standstill where the motive power is derived from electricity, in consequence of a breakdown in the supply.

The accommodation in the new buildings will be for 300 patients, 8 resident medical officers, 76 nurses, and 32 male and female attendants and servants. The wards in which these patients are to be accommodated are placed side by side, without any intervening space, practically under one roof, and of course without any side windows. Each ward is for 14 beds, and they are arranged in pairs, it being intended that each physician or surgeon shall have charge of a pair of wards, one male and the other female. Reference to the plan will show that each working unit consists of two main wards provided with closets, &c., at the far end and a bath room near the entrance, a kitchen common to both wards, a pair of separation wards, each containing two beds, linen cupboards and store for patients' clothes, and finally in the case of the surgical wards an operating room, and in that of the medical wards a clinical room; while for the use of the students in the medical side there are provided two large class rooms. Of these units there are eight, and in addition there is a ward for gynæcological cases, with its own separate kitchen, separation ward, and operating-room. These seventeen main wards, then, with their accessories, stand side by side, and trust entirely for air and heating to the air driven through them. At the south end of each main ward there is a large window opening on to a balcony, from which there is a fine view over park-like lands to the hills beyond, and from end to end of these wards there are lantern lights, glazed on the slightly sloping sides through which such sunlight as there may be can be admitted to the wards, or kept out by draw-down blinds, as may be desired. On the opposite side of the corridor from which all the above-mentioned wards open is an ophthalmic department, consisting of four wards (two for six and two for two beds) with a kitchen, an operating room, and a couple of bath rooms and closets; on this side of the corridor there are also the blocks for administration, an out-patient department, and entirely separated from the rest of the building there are two isolation blocks, each divided so as to accommodate both males and females. It is at once apparent that the compactness of the above arrangement of the wards not only simplifies administration and reduces labour to a minimum, but lends itself admirably to the details of the plenum system of ventilation, one great drawback to the adoption of which in hospitals of the pavilion plan is the length of the shafts or tunnels through which the air has to be carried for distribution. In the Belfast plan nothing can be simpler than the arrangement of the fresh air shafts supplying the wards. They can be kept perfectly clean, and are easily inspected from end to end. There is another point which seems to us to be worthy of consideration in this side-to-side construction, namely, that except for such heat as is lost through the windows at the end of each main ward, all the heat loss must take place through the roof and the lantern lights, that is at the top of the wards, the part where in all air-heated systems the heat tends most to accumulate. We do not wish to labour this point, but it is clear that in all cases where the heating of a building is by means of the air of ventilation it is important that loss of heat at or near the floor level shall be economised as far as possible. Now the economy of bottom heat from the absence of windows in this plan is obvious.

The appliances for securing ventilation are in a separate building at the east end of the main corridor, the principal air duct being 20 feet high by 9 feet wide. In this building the whole of the air supply for the hospital is to be drawn in, filtered through moistened screens, warmed in cold weather, and forced on by means of powerful air propellers in quantity sufficient to change the whole air contents of the hospital seven times per hour in winter, and ten times per hour in summer, without opening any windows. The appliances are all in duplicate, each of sufficient power to supply the minimum amount of air required, so that the necessary stoppages for cleansing, rest and repair of the machinery, may take place without stopping the air supply. It is stated by the architects that from the experience which they have acquired of plenum ventilation they have been able, in conjunction with Mr. Henry Lea, the consulting engineer, to simplify and cheapen the cost of the process, and that by utilising the exhaust steam from the engines, &c., they estimate that the cost incurred in maintaining the ventilation will not be much greater than that of the ordinary method. This of course is just what has to be proved. We may say, however, that the arrangements for carrying out the plenum system of warming and ventilation seem in this plan to be everything that can be desired. So much indeed is this the fact that it involves as a corollary the conclusion that by this hospital the plenum system will have to stand or fall, for if it does not succeed here it will hardly be likely to succeed elsewhere.

Reference to the plan will show a peculiarity, namely, the absence of all those cross-ventilated passages between wards and sanitary blocks which in hospitals ventilated in the ordinary way are generally regarded as essential. This is one of the facilities placed at the disposal of the architect by the plenum system. By the simple plan of placing an outlet over each sanitary block and so making the air pass continuously from the ward in that direction it is hoped that all reflux of air will be rendered impossible. We should add that the hospital stands upon a site of six acres; that the administrative buildings, of four stories and basement, in three separate blocks, on the north side of the main corridor, provide accommodation for the resident staff, the nurses' home, the attendants' and servants' quarters, and the dispensary and the kitchen departments; and that there will be a complete steam laundry, a disinfector, and a destructor, separated entirely from the hospital; as also will be the pathological department. The exterior of the building will be very simple, but from its elevation and the care which has been exercised in the selection of the materials it is hoped that it will be architecturally an acquisition to the city, notwithstanding that its cost, compared with many recently erected hospitals will be very low, namely, something not exceeding £300 per bed.

The architects are Mr. William Henman and Mr. Thomas Cooper, and the contract was let last autumn to Messrs. McLaughlin and Harvey, builders, of Belfast and Dublin, who have already made good progress with the works.

## Figures and Tables

**Figure f1:**